# Progression-Free Survival Early Assessment Is a Robust Surrogate Endpoint of Overall Survival in Immunotherapy Trials of Hepatocellular Carcinoma

**DOI:** 10.3390/cancers13010090

**Published:** 2020-12-30

**Authors:** Giuseppe Cabibbo, Ciro Celsa, Marco Enea, Salvatore Battaglia, Giacomo Emanuele Maria Rizzo, Anita Busacca, Domenica Matranga, Massimo Attanasio, Maria Reig, Antonio Craxì, Calogero Cammà

**Affiliations:** 1 Section of Gastroenterology & Hepatology, Department of Health Promotion, Mother and Child Care, Internal Medicine and Medical Specialties, PROMISE, University of Palermo, 90127 Palermo, Italy; giuseppe.cabibbo@policlinico.pa.it (G.C.); ciro.celsa@unipa.it (C.C.); giacomoemanuelemaria.rizzo@unipa.it (G.E.M.R.); anita.busacca@unipa.it (A.B.); antonio.craxi@unipa.it (A.C.); 2 Department of Surgical, Oncological and Oral Sciences (Di.Chir.On.S.), University of Palermo, 90127 Palermo, Italy; 3 Department of Health Promotion, Mother and Child Care, Internal Medicine and Medical Specialties, PROMISE, University of Palermo, 90127 Palermo, Italy; marco.enea@unipa.it (M.E.); domenica.matranga@unipa.it (D.M.); 4 Dipartimento di Scienze Economiche, Aziendali e Statistiche, University of Palermo, 90127 Palermo, Italy; salvatore.battaglia02@unipa.it (S.B.); massimo.attanasio@unipa.it (M.A.); 5 Barcelona Clínic Liver Cancer (BCLC) Group, Liver Unit, Hospital Clínic of Barcelona, IDIBAPS, CIBERehd, University of Barcelona, 08036 Barcelona, Spain; mreig1@clinic.cat

**Keywords:** hepatocellular carcinoma, immunotherapy, surrogate endpoints, progression-free survival, overall survival

## Abstract

**Simple Summary:**

Surrogate radiology-based endpoints such as progression-free survival (PFS) and objective response rate (ORR) are commonly used in oncology. However, their surrogacy with overall survival (OS) has not been evaluated in immunotherapy trials for hepatocellular carcinoma (HCC). We found that the surrogacy of PFS with OS is highly variable depending on treatment class (immune-checkpoint inhibitors or multikinase inhibitors) and evaluation time-point. Early PFS is a robust surrogate endpoint for OS in immunotherapy trials, while the surrogacy relationship between ORR and OS is weak. Early assessment of PFS could be useful for allowing analyses with small sample sizes and short accrual times, enhancing the interpretability of immunotherapy trials in HCC.

**Abstract:**

Background: Radiology-based outcomes, such as progression-free survival (PFS) and objective response rate (ORR), are used as surrogate endpoints in oncology trials. We aimed to assess the surrogacy relationship of PFS with overall survival (OS) in clinical trials of systemic therapies targeting advanced hepatocellular carcinoma (HCC) by novel meta-regression methods. Methods: A search of databases (PubMed, American Society of Clinical Oncology (ASCO), and European Society for Medical Oncology (ESMO) Meeting Libraries, Clinicaltrials.gov) for trials of systemic therapies for advanced HCC reporting both OS and PFS was performed. Individual patient data were extracted from PFS and OS Kaplan–Meier curves. Summary median PFS and OS data were obtained from random-effect model. The surrogate relationships of median PFS, first quartile (Q1), third quartile (Q3), and restricted mean survival time (RMST) for OS were evaluated by the coefficient of determination R^2^. Heterogeneity was explored by meta-regression. Results: We identified 49 trials, 11 assessing immune-checkpoint inhibitors (ICIs) and 38 multikinase inhibitors (MKIs). Overall, the correlation between median PFS and median OS was weak (R^2^ = 0.20. 95% Confidence Intervals [CI]-0.02;0.42). Surrogacy robustness varied between treatment classes and PFS endpoints. In ICI trials only, the correlations between Q1-PFS and Q1-OS and between 12-month PFS-RMST and 12-month OS-RMST were high (R^2^ = 0.89, 95%CI 0.78–0.98, and 0.80, 95% CI 0.63–0.96, respectively). Interaction *p*-values obtained by meta-regression confirmed the robustness of results. Conclusions: In trials of systemic therapies for advanced HCC, the surrogate relationship of PFS with OS is highly variable depending on treatment class (ICI or MKI) and evaluation time-point. In ICI trials, Q1-PFS and 12-month PFS-RMST are robust surrogate endpoints for OS.

## 1. Introduction

Hepatocellular carcinoma (HCC) is often diagnosed at advanced stage not amenable to curative treatments [1]. In recent years, there has been a surge in progress for HCC treatment, leading to the development of several systemic therapies [2,3]. Given the rapid ongoing evolution in this area, a careful evaluation of trial designs and outcomes to optimize health benefits to patients is needed.

Overall survival (OS) is a universally recognized easy-to-assess endpoint to determine clinical benefit in oncology trials [4]. However, the interpretation of OS can be confounded by post-progression survival and treatment crossover [5]. Surrogate radiology-based endpoints, such as progression-free survival (PFS), time-to-progression (TTP), and objective response rate (ORR), are commonly used in oncology, especially when sequential post-progression treatments are available, as is now occurring for HCC [6]. Their relevance remains debated, and aggregate-data meta-analyses showed a modest correlation with OS, with substantial variability according to cancer type and stage and according to the class of drug(s) administered [7]. Specifically, PFS is a composite endpoint that might provide an early assessment of treatment efficacy, independent of post-progression survival [5]. However, PFS is limited by the subjectivity inherent in radiological evaluation of progression and using different response criteria [8]. A recent meta-analysis of aggregated data from randomized controlled trials (RCTs) of systemic therapies for HCC, not including immunotherapy drugs, showed only a moderate correlation between PFS and OS [9]. Meanwhile, for immunotherapy trials involving patients with different types of cancer, a weak association was found between PFS and OS both at individual level and trial level [10,11]. 

Here, we report a meta-regression of clinical trials of systemic therapies, including immunotherapies, for advanced HCC. The aim of this meta-regression was to evaluate the surrogate relationship between radiology-based endpoints (PFS and ORR) and OS.

## 2. Results

### 2.1. Trial Selection and Characteristics

Trial selection process is showed in Figure S1. Based on the full-text reviews, we determined that 49 clinical trials fulfilled the inclusion criteria, and these trials were selected for the main analysis.

The characteristics of the 49 studies in the main analysis, including a total of 85 arms and 15,038 patients, are summarized in Table S1. There were 11 trials assessing immune-checkpoint inhibitors (ICIs) and 38 assessing multikinase inhibitors (MKIs) (Table 1 and Table S2, respectively). Data on median PFS were available in 35 trials (44 treatment arms), including 8 assessing ICIs (9 arms) and 27 assessing MKIs (35 arms). ORR data were available for 36 trials (62 arms), 11 assessing ICIs (14 arms) and 35 assessing MKIs (48 arms).

**Figure 1:**
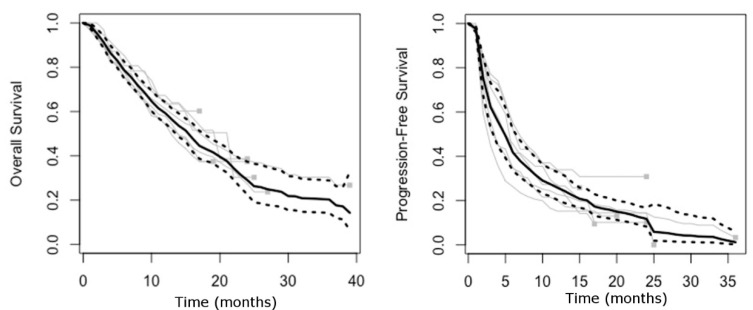
Pooled reconstructed survival curves for overall survival (OS) and progression-free survival (PFS) from clinical trials assessing immune-checkpoint inhibitors (ICIs) in advanced hepatocellular carcinoma (HCC). Black lines represent pooled reconstructed survival curves; dotted lines represent lower and upper 95% confidence interval; gray lines represent reconstructed survival curves of single trial arms.

Individual patient data (IPD) for PFS and OS were extracted from 24 trials (30 arms including 7104 patients), including 6 trials evaluating ICIs (7 arms including 1377 patients) and 18 evaluating MKIs (23 arms including 5727 patients), to obtain reconstructed survival curves. As shown in Figure 1, in ICI trials, the OS pooled median was 15.4 months (95% Confidence Interval [95%CI] 13.3–17.1. First quartile [Q1]-Third quartile [Q3]: 6.9–36.3. I^2^ = 0%) and the PFS pooled median was 4.9 months (95%CI 3.2–6.1. Q1–Q3: 1.9–11.2. I^2^ = 43.6%). In first-line ICI trials, OS and PFS pooled median were 17.6 (95%CI 14.6–19.4) and 5.5 months (95%CI 2.8–7.7), respectively. In second-line ICI trials OS and PFS pooled median were 13.5 months (95%CI 11.9–15.0) and 3.5 months (95%CI 2.4–5.2), respectively (Figures S2 and S3).

For MKI trials, the OS pooled median was 9.9 months (95%CI 9.0–11.2. Q1–Q3: 5.2–21.7. I^2^ = 37.6%) and the PFS pooled median was 3.9 months (95%CI 3.3–4.9. Q1–Q3: 1.9–7.9. I^2^ = 67.6%) (Figure S4). In first-line MKI trials, OS and PFS pooled median were 9.9 (95%CI 8.7–11.4) and 4.3 months (95%CI 3.4–5.3), respectively. In second-line MKI trials OS and PFS pooled median were 9.9 months (95%CI 8.6–11.7) and 3.4 months (95%CI 2.7–4.3), respectively (Figures S5 and S6).

Restricted mean survival times (RMSTs) for each trial are reported in Table 2. Six-month OS and PFS RMSTs were 5.5 and 4.0 months for ICI trials and 5.3 and 3.9 months for MKI trials, respectively. Twelve-month OS and PFS RMSTs were 9.5 and 5.8 months for ICI trials and 8.6 and 5.2 months for MKI trials, respectively. 

**Figure 2:**
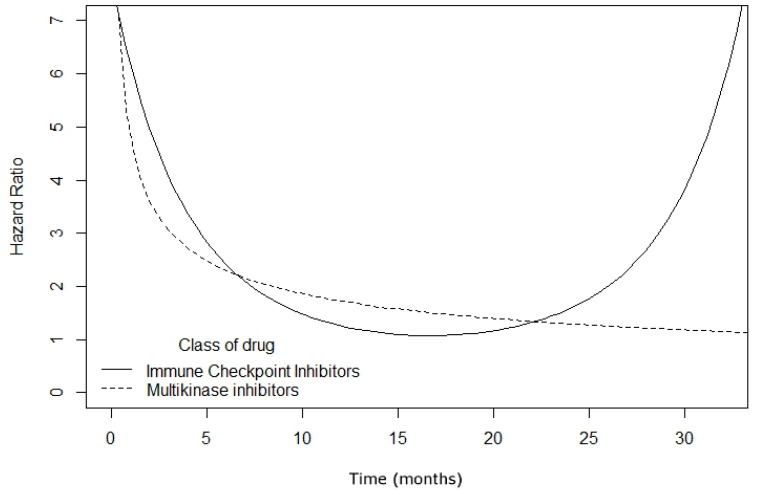
Estimated hazard ratios (HRs) over time between PFS and OS in clinical trials of ICIs and multikinase inhibitors (MKIs) for advanced HCC.

Non-proportionality of hazards between PFS and OS was present in 66.7% of the treatment arms (71.4% of ICI-arms; 65.2% of MKI-arms) (Table S3). Time-dependent Cox modeling confirmed the non-proportionality of hazards between PFS and OS in pooled reconstructed survival curves, showing that hazards vary over time, following a different trend in ICIs than in MKIs (Figure 2, Table S4, Figure S7).

### 2.2. Surrogacy Metrics

**Figure 3:**
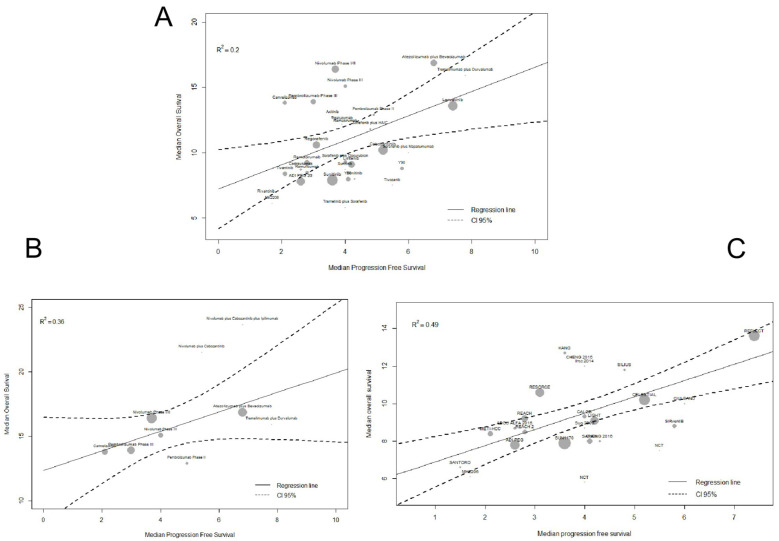
Meta-regression analysis of the relationship between median PFS and median OS in clinical trials of systemic therapies for advanced HCC. (**A**) Overall. (**B**) ICI trials. (**C**) MKI trials.

As we verified that hazards were not proportional, we assessed the surrogacy relationship between PFS and OS by analyzing median times, time-based endpoints (Q1 and Q3), and RMSTs. In the weighted linear regression between median OS and median PFS, the R^2^ values were 0.20 (95%CI-0.02;0.42) overall, 0.36 (95%CI 0.02;0.70) for ICI trials, and 0.49 (95%CI 0.24;0.73) for MKI trials (Figure 3).

Regarding time-based PFS and OS endpoints, the overall R^2^ values were 0.43 (95%CI 0.19;0.67) between Q1-PFS and Q1-OS and 0.17 (95%CI −0.05;0.38) between Q3-PFS and Q3-OS (Figure S8). For ICI trials, the Q1 and Q3 R^2^ were 0.89 (95%CI 0.78;0.98) and 0.08 (95%CI −0.15;0.32), respectively (Figure S9). For MKI trials, the Q1 and Q3 R^2^ values were 0.46 (95%CI 0.20;0.72) and 0.50 (95%CI 0.25;0.75) respectively (Figure S10). 

**Figure 4:**
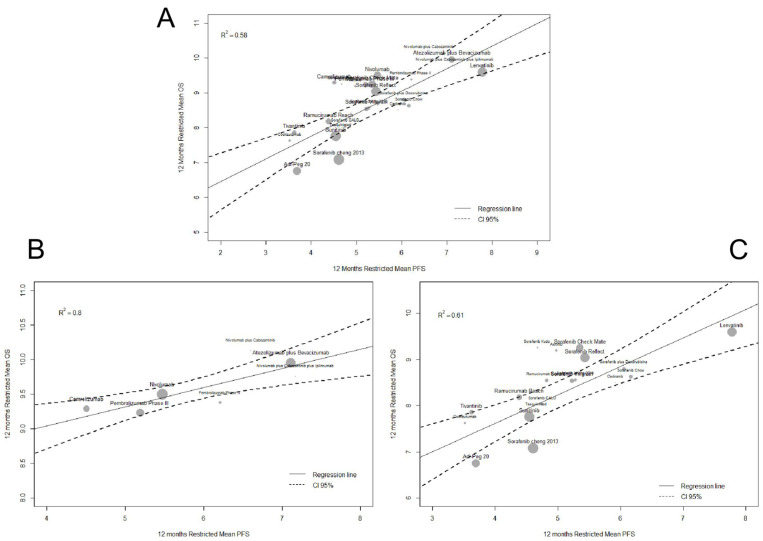
Meta-regression analysis of the relationship between 12-month restricted mean survival times (RMSTs) for PFS and OS in clinical trials of systemic therapies for advanced HCC. (**A**) Overall. (**B**) ICI trials. (**C**) MKI trials.

In the weighted linear regression between 6-month PFS and OS RMSTs, the R^2^ values were 0.50 (95%CI 0.25;0.75) overall, 0.80 (95%CI 0.64;0.96) for ICI trials, and 0.55 (95%CI 0.30;0.80) for MKI trials (Figure S11). Between 12-month PFS and OS RMSTs, the R^2^ values were 0.58 (95%CI 0.36;0.79) overall, 0.80 (95%CI 0.63;0.96) for ICI trials, and 0.61 (95%CI 0.38;0.83) for MKI trials (Figure 4). The results of these analyses are also reported in Table S5.

The results of subgroup analyses and p-values for interaction are reported in Table S6 and Figures S12–S15. 

Surrogacy analysis between ORR and OS rate at the end of follow-up yielded overall, ICI-trial, and MKI-trial R^2^ values of 0.005 (95% CI −0.03;0.04), 0.60 (95% CI 0.33;0.88), and 0.002 (95% CI −0.0008;0.0009), respectively (Figure S16). OS rates at the end of follow-up and the results of subgroup analyses for ORR are reported in Tables S7 and S8, respectively.

## 3. Discussion

To the best of our knowledge, this is the first systematic quantitative study that assessed the correlation between surrogate and true treatment endpoints in trials of systemic therapies for advanced HCC by using innovative methods. Median PFS and median OS were found to be weakly correlated. Surrogacy relationships among outcomes varied according to treatment class (MKI or ICI) and PFS evaluation time-point. In ICI trials, but not MKI trials, the surrogacies between Q1-PFS and Q1-OS and between 12-month PFS-RMST and 12-month OS-RMST were high. ORR could not be confirmed as a robust surrogate endpoint for OS. 

Innovative methodologies aimed at validating the role of radiology-based outcomes (TTP, PFS, and ORR) as surrogate endpoints of OS are becoming increasingly relevant in oncology. In particular, the advent of ICIs for the treatment of HCC has raised several questions regarding the most appropriate surrogate endpoints for early capture of survival benefit. Consequently, validated and consistent new methodological criteria for defining response to treatment are urgently needed. PFS is a composite endpoint not influenced by post-progression survival and that avoids crossover treatment bias. Modeling sequential treatments, PFS represents the primary endpoint for first-line therapy in ICI trials, as demonstrated by a recently published decision model [40]. Although efforts have been made to improve the surrogacy between PFS and OS in immunotherapy trials—by modifying the threshold percentage to define PFS or the response criteria with Immune Response Evaluation Criteria in Solid Tumors (iRECIST)—PFS surrogacy for OS remains weak both at trial and individual level [10,11,41]. Therefore, the potential for alternative treatment effect measures (Q1-PFS, RMST, and milestone analysis) able to early capture survival benefits, while traditional statistical methods (medians, hazard ratios [HRs], log rank tests) cannot, is a key issue in the era of immunotherapy [42]. Although HR is the most commonly used comparative measure, its validity is limited by the requirement to assume a proportional hazard over the entire follow-up period [43,44]. Upon demonstrating that this assumption did not hold between PFS and OS for both ICI- and MKI trials, we explored whether this surrogate relationship can be improved by adopting new robust statistical procedures. First quartile analysis is a cross-sectional assessment of treatment benefit at a meaningful time-point that overcomes the proportional hazards assumption. Our analysis showed that the surrogacy between Q1-PFS and Q1-OS was high in ICI trials only. However, these time-based outcomes do not reflect the entire survival history. Overcoming this limitation, RMST represents an innovative methodology that has the advantage of being valid under any time-to-event distribution, regardless of the proportional hazard assumption [42,43,44]. Unlike HR, RMST is an absolute measure of survival time, it can be used in all models, and it does not change with extended follow-up, enabling clinically meaningful interpretation of a treatment effect [44,45]. Although it was intended to increase the interpretability of immunotherapy trials, it is not routinely reported.

In ICI trials, our analyses showed that PFS surrogacy of OS was robust with the use of 12-month RMSTs. In particular, we further confirmed the significant benefit of atezolizumab plus bevacizumab compared to sorafenib, both for 12-month RMST OS and PFS, when we reanalyzed data from the recently published RCT [2]. Moreover, our data may have important implications also for trial design and for sample size calculation of future ICI trials.

Importantly, our results suggest that this surrogate relationship changes over time, and that these changes follow different trends for ICI trials than for MKI trials. In MKI setting, caution must be taken when interpreting PFS in absence of OS. The reasons underlying this finding are not fully understood, but they could be plausibly related to different pharmacodynamics between MKIs (fast) and ICIs (slow but durable). Therefore, we can hypothesize that the durable radiological response to ICIs better correlates with OS [46].

It is important to consider that the line of treatment could have an impact on the surrogacy between PFS and OS, because patients on first-line treatment are more likely to have a chance to receive subsequent post-progression treatments compared to patients on second-line treatment. Unfortunately, the small number of first-line ICI trials [2,17] hampered this subgroup analysis.

Our meta-regression demonstration of a weak correlation between ORR and OS underscores that researchers should exercise caution when using the ORR as the primary endpoint in a phase III trial of an immunotherapy, with deference being given to time-to-event outcomes (e.g., PFS, TTP, time to response, and duration of response). The correlation value obtained is consistent with the results of a prior meta-analysis of immunotherapy trials conducted in other cancer types, such as melanoma, lung cancer, and renal cell carcinoma [10] and with an aggregate-data meta-analysis including only MKI RCTs of HCC [9]. Together, this convergence of evidence does not lend support the use of the ORR as a primary endpoint in immunotherapy trials. Accordingly, treatment effects based solely on time-fixed surrogate outcomes, such as ORRs, should be interpreted with caution.

Limitations: Although we extracted IPD for OS and PFS from Kaplan–Meier curves, the association between PFS and OS could not be evaluated at the individual level. Moreover, we were unable to assess other potentially relevant patient-level covariates, such as duration of response, treatment-related toxicity, and hepatic decompensation. The survival of patients with advanced HCC has been shown to be influenced by hepatic decompensation, which, together with HCC progression, represents a competitive mortality risk [47]. Finally, we agree fully with Finn that an IPD meta-analysis could better evaluate the surrogacy between PFS and OS [6].

## 4. Materials and Methods

### 4.1. Literature Search and Study Selection

Details about literature search are reported in Supplementary Materials. 

The inclusion criteria for retrieved studies were: being a clinical trial of systemic therapy for advanced HCC; and data reported for OS and at least one surrogate radiology-based endpoint (PFS or ORR). Review articles, letters, interim analyses, subgroup analyses of previously reported trials, trials including only conventional chemotherapy, duplicate reports, trials in which the systemic therapy of interest was used in an adjuvant and neoadjuvant setting, or used with concomitant locoregional treatments were excluded. Each trial was evaluated by three independent investigators (Ci.C., G.E.M.R., A.B.). Discrepancies among reviewers were not frequent (interobserver variation < 10%) and resolved by discussion.

### 4.2. Trial-Level Data Extraction 

OS/PFS median times and HRs with corresponding 95% CIs and ORRs were assessed as measures of treatment effect. We also obtained the following covariates: ICI or MKI treatment; single-agent or combination therapy; trial phase; publication year; number of trial arms; number of patients in each arm; type of control arm; treatment line; timing of first radiological assessment; follow-up duration; and treatment-response radiological evaluation criteria.

### 4.3. Individual Patient Survival Data Extraction

We used Engauge Digitizer software [48] to extract IPD from OS and PFS Kaplan–Meier curves and used Guyot algorithm [49] to reconstruct the data. This algorithm was applied to assembled patients with predicted survival times and a predicted event of interest (i.e., alive or dead; progression or no progression) with digitized data on survival probabilities, time, and total numbers of patients and events. Each reconstructed survival curve was inspected for accuracy and compared with the originally published curves. 

We used Combescure [50] nonparametric approach to obtain summary survival curves, which enabled assessments of pooled reconstructed survival probabilities of trials separately according to drug class (ICI or MKI). A random-effects model was used to detect between-study heterogeneity. The multivariate extension of DerSimonian and Laird’s method was used to estimate a between-study covariance matrix [51,52]. Heterogeneity was assessed by the I^2^ statistic.

### 4.4. Restricted Mean Survival Time (RMST)

RMSTs, reflecting average survival from time 0 to a specified time-point t, were determined from Kaplan–Meier estimates of survival functions. RMST can be interpreted readily as the area under the survival curve within a specific time window. For each trial, we reanalyzed the reconstructed IPD and then assessed RMSTs for OS and PFS at two pre-specified time horizons: 6 and 12 months [53].

### 4.5. Statistical Analysis

We used a two-step process to evaluate the surrogate relationship between PFS and OS.

#### 4.5.1. Step 1: Assessing Proportional Hazards Assumption

We first checked if the proportional hazards (PH) assumption between PFS and OS was valid in each trial and in the pooled PFS and OS curves for each drug class (ICI or MKI) using Schoenfeld residual statistics. When the PH assumption was not verified, we generated time-dependent Cox models, including an interaction term between survival time and the fixed covariate, to overcome the non-proportionality [54]. The best model was chosen based on Akaike’s information criterion values.

#### 4.5.2. Step 2: Surrogacy Endpoint Validation

Linear meta-regression model, with sample-size weighting of the trial arms from which the data were extracted, was employed to quantify the relationship between PFS and OS. Surrogacy was evaluated between median times, between different time-based endpoints [first quartile (Q1) and third quartile (Q3)], between 6-month RMSTs, and between 12-month RMSTs. For ORR surrogacy validation, we assessed the relationship between OS rate at the end of follow-up and ORR (not being this latter a time-to-event endpoint). The strength of each association was assessed by calculating R^2^ (the proportion of OS variance that is predictable from the surrogate endpoints), with values near 1 implying surrogacy and values close to zero suggesting no association [55].

### 4.6. Subgroup Analyses 

We performed the following subgroup analyses: (1) drug class (ICI or MKI); (2) presence of control arm (controlled or not controlled); (3) trial phase (phase I/II or III); (4) line of treatment (first or second); and (5) duration of follow-up. For each subgroup analysis, we calculated an interaction *p*-value using a meta-regression model.

## 5. Conclusions

In trials of systemic therapies for advanced HCC, the surrogacy relationship of PFS with OS is highly variable depending on treatment class (ICI or MKI) and evaluation time-point. In ICI trials, Q1-PFS and 12-month PFS-RMST are robust surrogate endpoints for OS. Therefore, PFS RMSTs should be reported routinely in ICI trials for advanced HCC. Although caution must be taken when interpreting PFS in the absence of OS data, PFS could be useful for allowing analyses with small sample sizes and short accrual times in clinical trials, ultimately enhancing the interpretability of immunotherapy clinical trials.

## Figures and Tables

**Table 1:** Characteristics and clinical outcomes of ICI clinical trials for the treatment of advanced hepatocellular carcinoma (HCC) included in the study.

	**Trial**	**Line of Treatment**	**Arms**	**Overall Survival**				**Progression-Free Survival**				**Objective Response Rate (%)**	**Time to First Radiological Assessment**	**Duration of Follow-Up** **(Months)**	**Reference**
				**1st Quartile (Months)**	**Median (Months)**	**3rd Quartile (Months)**	**HR (95% CI)**	**1st Quartile (Months)**	**Median (Months)**	**3rd Quartile (Months)**	**HR (95% CI)**				
**Immune-checkpoint Inhibitors Alone Or In Combination**	KEYNOTE-240, 2019 (phase III, full text)	Second-line	Pembrolizumab (*n* = 278)	6.15	13.9	24	0.781 (0.611–0.998)	1.46	3.0	8.45	0.718 0.570–0.904)	18.3	6 weeks	28	[12]
			Placebo (*n* = 135)	-	10.6	-		-	2.8	-		4.4			
	KEYNOTE-224, 2018 (phase II, full text)	Second-line	Pembrolizumab (*n* = 104)	7.25	12.9	16.1	-	2.12	4.9	12.5	-	17.3	9 weeks	19	[13]
	CheckMate 040, 2017 (phase I/II, full text)	Second-line	Nivolumab (*n* = 182)	-	15.1	-	-	-	4.0	-	-	14.3	-	57	[14]
	Qin et al., 2020 (phase II, full text)	Second-line	Camrelizumab (*n* = 217)	6.45	13.8	16.9	-	1.88	2.1	6.1	-	14.7	8 weeks	22	[15]
	Sangro et al., 2013 (phase II, full text)°	Both first- and second-line	Tremelimumab (*n* = 21)	6	8.2	21.6	-	-	NA		-	17.6	12 weeks	25	[16]
	CheckMate 459, 2019 (phase III abstract)	First-line	Nivolumab (*n* = 371)	6.35	16.4	36.3	0.85 (0.72–1.02)	1.98	3.7	9.90	0.93 (0.79–1.10)	15.4	-	39	[17]
			Sorafenib (*n* = 372)	6.0	14.7	27.3		1.95	3.8	7.65		7.0		37	
	NCT01693562, 2017 (phase I/II, abstract)	Second-line	Durvalumab (*n* = 39)	-	13.2	-	-	-	NA	-	-	10.3	-	-	[18]
	CheckMate 040, 2019 (phase I/II, abstract)	Second-line	Nivolumab plus Ipilimumab * Arm A: *n* = 50	-	Arm A: 23	-	-	-	NA	-	-	Arm A: 32	-		
			Arm B: *n* = 49	-	Arm B: 12	-		-		-		ArmB:30.6		37	[19]
			Arm C: *n* = 49	-	Arm C: 13	-		-		-		Arm C: 30.6			
	NCT02821754, 2019 (phase I/II, abstract)	Second-line	Tremelimumab plus Durvalumab (*n* = 10)	-	15.9	-	-	-	7.8	-	-	20	-	-	[20]
**Immune-checkpoint inhibitors in combination with multikinase inhibitors**	CheckMate 040, 2020 (phase I/II, abstract)	Both first- (*n* = 12) and second-line (*n* = 19)	Nivolumab plus Ipilimumab plus Cabozantinib (*n* = 35)	9	24	NR	-	3.4	6.8	NR	-	31	-	24	[21]
		Both first- (*n* = 12) and second-line (*n* = 23)	Nivolumab plus Cabozantinib (*n* = 36)	8.7	21.5	NR	-	2.73	5.4	12.5	-	14			
	IMbrave 150, 2019 (phase III, published)	First-line	Atezolizumab plus Bevacizumab (*n* = 336)	7.87	NR	NR	0.58(0.42–0.79)	2.9	6.8	13.5	0.59 (0.47–0.76)	26.5	6 weeks	17	[2]
			Sorafenib (*n* = 165)	3.5	13.2	-		1.17	4.3	7.3		11.9			

The treatment arms from which the individual data for overall survival (OS) and progression-free survival (PFS) were extracted are shown in bold. All the included trials employed Response Evaluation Criteria in Solid Tumours (RECIST) 1.1. * Arm A: NIVO 1 mg/kg + IPI 3 mg/kg Q3W (4 doses), followed by NIVO 240 mg Q2W. Arm B: NIVO 3 mg/kg + IPI 1 mg/kg Q3W (4 doses), followed by NIVO 240 mg Q2W. Arm C: NIVO 3 mg/kg Q2W + IPI 1 mg/kg Q6W. HR, Hazard Ratio. 95% CI, 95% Confidence intervals. NA, not available. NR, not reached.

**Table 2:** Six-month and twelve-month restricted mean survival times (RMSTs) for progression-free survival (PFS) and overall survival (OS) in clinical trials of advanced HCC.

Class of Drug	Trial Arm	6-Month Restricted Mean Survival Time		12-Month Restricted Mean Survival Time	
		Progression-Free Survival (Months) (95% Confidence Interval)	Overall Survival (Months) (95% Confidence Interval)	Progression-Free Survival (Months) (95% Confidence Interval)	Overall Survival (Months) (95% Confidence Interval)
**Immune-checkpoint inhibitors**	IMBrave-150 (Atezolizumab plus Bevacizumab arm) [2]	4.67 (4.46–4.86)	5.62 (5.50–5.73)	7.11 (6.65–7.58)	9.95 (9.60–10.31)
	CheckMate 459 (Nivolumab arm) [17]	3.85 (3.35–4.04)	5.42 (5.28–5.56)	5.47 (5.03–5.9)	9.50 (9.12–9.87)
	Keynote-240 (Pembrolizumab arm) [12]	3.65 (3.40–3.89)	5.37 (5.22–5.52)	5.19 (4.68–5.70)	9.23 (8.79–9.66)
	Keynote-224 (Pembrolizumab arm) [13]	4.23 (3.87–4.59)	5.44 (5.19–5.69)	6.21 (5.41–7.02)	9.38 (8.70–10.05)
	Qin et al., 2020 (Camrelizumab arm) [15]	3.32 (3.07–3.57)	5.35 (5.16–5.23)	4.51 (3.99–5.04)	9.29 (8.79–9.79)
	CheckMate 040 (Nivolumab plus Cabozantinib plus Ipilimumab arm) [21]	4.72 (4.13–5.32)	5.37 (4.84–5.89)	7.17 (5.77–8.57)	9.76 (8.38–11.41)
	CheckMate 040 (Nivolumab plus Cabozantinib arm) [21]	4.44 (3.84–5.03)	5.62 (5.31–5.93)	6.6 (5.2–8)	10.13 (9.02–11.24)
Pooled immune-checkpoint inhibitors		3.99 (3.88–4.09)	5.45 (5.38–5.52)	5.79 (5.56–6.02)	9.53 (9.34–9.72)
**Multikinase inhibitors**	SUN1170 (Sorafenib arm) [22]	3.52 (3.35–3.69)	4.81 (4.56–5.07)	4.61 (4.28–7.93)	7.08 (6.44–7.71)
	SUN1170 (Sunitinib arm) [22]	3.68 (3.53–3.84)	5.00 (4.87–5.13)	4.54 (4.26–4.82)	7.76 (7.42–8.09)
	SARAH (Sorafenib arm) [23]	3.91 (3.65–4.18)	5.21 (5.01–5.4)	5.22 (4.71–5.72)	8.54 (8.02–9.06)
	SIRVENIB (Sorafenib arm) [24]	4.48 (4.25–4.72)	5.28 (5.06–5.49)	6.16 (5.59–6.72)	8.63 (8.05–9.21)
	CALGB80802 (Sorafenib arm) [25]	3.68 (3.39–3.97)	5.00 (4.70–5.24)	4.75 (4.20–5.30)	8.03 (7.44–8.63)
	SILIUS (Sorafenib arm) [26]	3.71 (3.35–4.07)	5.5 (5.39–5.59)	4.68 (4.00–5.36)	9.25 (8.93–9.56)
	Hsu et al., 2012 (Vandetanib arms) [27]	2.19 (1.4–2.97)	4.86 (4.17–5.56)	NA	NA
	Abou-Alfa et al., 2010 (Sorafenib plus Doxorubicin arm) [28]	4.38 (3.79–4.98)	5.15 (4.66–5.63)	6.05 (4.85–7.25)	8.8 (7.6–10.00)
	REFLECT (Sorafenib arm) [29]	3.96 (3.77–4.14)	5.43 (5.32–5.54)	5.43 (5.05–5.81)	9.04 (8.71–9.36)
	REFLECT (Lenvatinib arm) [29]	4.85 (4.67–5.00)	5.54 (5.43–5.64)	7.78 (7.38–8.16)	9.29 (9.6–9.91)
	IMBrave-150 (Sorafenib arm) [2]	3.96 (3.64–4.28)	5.25 (5.13–5.37)	5.28 (4.65–5.91)	8.56 (8.23–8.89)
	CheckMate 459 (Sorafenib arm) [17]	4.02 (3.83–4.21)	5.40 (5.27–5.54)	5.35 (4.96–5.74)	9.25 (8.87–9.63)
	Zhu et al., 2013 (Cediranib arm) [30]	4.29 (3.39–5.19)	5.15 (4.30–6.00)	5.92 (4.12–7.72)	8.50 (6.51–10.50)
	RESORCE (Regorafenib arm) [31]	3.71 (3.51–3.91)	5.22 (5.08–5.36)	4.89 (4.49–5.29)	8.63 (8.23–9.03)
	CELESTIAL (Cabozantinib arm) [32]	4.19 (4.01–4.36)	5.30 (5.16–5.44)	5.29 (5.66–6.03)	8.76 (8.39–9.12)
	REACH (Ramucirumab arm) [33]	3.31 (2.00–3.61)	5.17 (4.97–5.37)	4.39 (3.84–4.94)	8.18 (7.64–8.72)
	REACH-2 (Ramucirumab arm) [34]	3.54 (3.30–3.79)	5.26 (5.09–5.42)	4.82 (4.35–5.29)	8.55 (8.09–9.01)
	METIV-HCC (Tivantinib arm) [35]	3.08 (2.62–3.15)	4.99 (4.79–5.19)	3.62 (3.25–3.99)	7.86 (7.34–8.38)
	ADI-PEG20 (ADI-PEG20 arm) [36]	3.19 (3.01–3.37)	4.47 (4.29–4.65)	3.69 (3.40–3.98)	6.75 (6.34–7.16)
	Kang et al., 2015 (Axitinib arm) [37]	3.75 (3.41–4.09)	5.37 (4.84–5.89)	4.98 (4.32–5.62)	9.19 (8.56–9.82)
	Escudier et al., 2017 (Tasquinimod arm) [38]	3.73 (3.20–4.27)	5.30 (4.88–5.72)	4.65 (3.69–5.61)	7.89 (6.89–8.89)
	Abou-Alfa et al., 2016 (Codrituzumab arm) [39]	3.02 (2.68–3.36)	4.97 (4.68–5.26)	3.52 (2.97–4.07)	7.62 (6.96–8.29)
Pooled multikinase inhibitors		3.86 (3.80–3.90)	5.26 (5.22–5.30)	5.19 (5.08–5.30)	8.63 (8.53–8.73)

## Data Availability

The data presented in this study are available on request from the corresponding author.

## Supplementary Materials

The following are available online at https://www.mdpi.com/2072-6694/13/1/90/s1, Literature search. Table S1: Characteristics of eligible trials evaluating immune-checkpoint inhibitors (ICIs) and multikinase inhibitors (MKIs) (*n* = 49), Table S2: Characteristics and clinical outcomes of the multikinase inhibitor (MKI) clinical trials for advanced hepatocellular carcinoma (HCC) included in the study, Table S3: Test of proportionality of hazards between PFS and OS in reconstructed survival curves for ICIs and multikinase inhibitors (MKIs), Table S4: Time functions of the best time-dependent Cox model for each drug, Table S5: Results of weighted linear meta-regression between PFS and OS in clinical trials of systemic therapies for advanced HCC, Table S6: Results of subgroup analyses of surrogacy between PFS and OS, Table S7: OS rate at the end of follow-up in clinical trials of systemic therapies for advanced HCC, Table S8: Results of subgroup analyses of surrogacy between objective response rate (ORR) and OS rate at the end of follow-up, Figure S1: Study flow chart, Figure S2: Pooled reconstructed survival curves of overall survival (OS) of clinical trials assessing ICIs in advanced hepatocellular carcinoma (HCC) in first- (**A**) and second-line (**B**) trials, Figure S3: Pooled reconstructed survival curves of progression-free survival (PFS) of clinical trials assessing ICIs in advanced hepatocellular carcinoma (HCC) in first- (**A**) and second-line (**B**) trials, Figure S4: Pooled reconstructed survival curves of OS (**A**) and PFS (**B**) of clinical trials assessing multikinase inhibitors (MKIs) in advanced hepatocellular carcinoma (HCC), Figure S5: Pooled reconstructed survival curves of OS of clinical trials assessing multikinase inhibitors (MKIs) in advanced hepatocellular carcinoma (HCC) in first- (**A**) and second-line (**B**) trials, Figure S6: Pooled reconstructed survival curves of PFS of clinical trials assessing multikinase inhibitors (MKIs) in advanced hepatocellular carcinoma (HCC) in first- (**A**) and second-line (**B**) trials, Figure S7: Trend of beta coefficient of treatment over time in clinical trials of ICIs (**A**) and MKIs (**B**) for advanced HCC, Figure S8: Meta-regression analysis of the relationship between the time-based endpoints of PFS and OS in clinical trials of systemic therapy for advanced HCC treatment. (**A**) First quartile. (**B**) Third quartile, Figure S9: Meta-regression analysis of the relationship between the time-based endpoints of PFS and OS in clinical trials of ICIs for advanced HCC treatment. (**A**) First quartile. (**B**) Third quartile, Figure S10: Meta-regression analysis of the relationship between the time-based endpoints of PFS and OS in clinical trials of MKIs for advanced HCC treatment. (**A**), First quartile. (**B**), Third quartile, Figure S11: Meta-regression analysis of the relationship between 6-month PFS and OS restricted mean survival times (RMSTs) in clinical trials of systemic therapies for advanced HCC. (**A**) Overall. (**B**) ICI trials. (**C**) MKI trials, Figure S12: Meta-regression analysis of the relationship between PFS and OS in controlled (**A**) and uncontrolled (**B**) clinical trials of systemic therapies for advanced HCC using median times, Figure S13: Meta-regression analysis of the relationship between PFS and OS in phase III trials (**A**) and in phase II and I/II trials (**B**) of systemic therapies for advanced HCC using median times, Figure S14: Meta-regression analysis of the relationship between PFS and OS in first-line trials (**A**) and second-line trials (**B**) of systemic therapies for advanced HCC using median times, Figure S15: Meta-regression analysis of the relationship between PFS and OS in clinical trials of systemic therapies for advanced HCC with duration of follow-up longer than 30 months (**A**) and shorter than 30 months (**B**), using median times, Figure S16: Meta-regression analysis of the relationship between ORR and OS in clinical trials of systemic therapies for advanced HCC. (**A**) Overall. (**B**) ICIs. (C) MKIs.
