# Supplementary material for: Progression-Free Survival Early Assessment Is a Robust Surrogate Endpoint of Overall Survival in Immunotherapy Trials of Hepatocellular Carcinoma
Source: Cancers (Basel). 2020 Dec 30;13(1):90. doi: 10.3390/cancers13010090 (PMC7796103; doi:10.3390/cancers13010090)
Supplement: Supplementary file 1 [file cancers-13-00090-s001.pdf]

Supplementary materials

Literature search

The MEDLINE database was searched systematically up to June 5, 2020 with the search terms "advanced hepatocellular carcinoma" and "clinical trial". Moreover, we searched ClinicalTrials.gov for trials with results posted (Studies with results) using "hepatocellular carcinoma unresectable", "hepatocellular carcinoma metastatic" and "hepatocellular carcinoma stage IV" as keywords. Search criteria included interventional studies and phase I to IV. We also searched abstracts presented in the ASCO (American Society of Clinical Oncology) Meeting Library and ESMO (European Society for Medical Oncology) Conference Platform during the last 5 years. Abstracts published subsequently as full-text studies already included in our analysis were excluded.

Supplementary Table

Table S1. Characteristics of eligible trials evaluating immune checkpoint inhibitors (ICIs) and multikinase inhibitors (MKIs) (n= 49)

Characteristics	Overall (n= 49)	Immune check point inhibitors (n=11)	Multikinase inhibitors (n = 38)
**Phase of trials**			
Phase III	22	3	19
Phase II	20	3	17
Phase I/II	7	5	2
**Publication status**			
Published	34	5	29
Unpublished	15	6	9
**Arms in each trial**			
3	3	1	2
2	29	4	25
1	17	6	11
**Treatment line**			
First-line	26	2	24
Second-line	20	7	13
Both first- and second-line	3	2	1

**Treatment**			
Single agent	37	7	30
Combination therapy	12	4	8
**Controls**			
Placebo	15	1	14
Sorafenib	14	2	12
Others*	3	2	1
Uncontrolled	17	6	11

*****For ICIs, the other control arms are represented by Nivolumab plus Cabozantinib and by Nivolumab plus Ipilimumab at different dosages. For MKIs, the other control arm is represented by Doxorubicin.

Table S2. Characteristics and clinical outcomes of the multikinase inhibitor (MKI) clinical trials for advanced hepatocellular carcinoma (HCC) included in the study.

Trial	Line of treatment	Arms	Overall Survival				Progression-free Survival				Objective Response Rate (%)	Time to first radiological assessment	Duration of follow-up (months)	Reference
			1st Quartile (months)	Median (months)	3rd Quartile (months)	HR (95% CI)	1st Quartile (months)	Median (months)	3rd Quartile (months)	HR (95% CI)				
SHARP, 2008 (phase III, full text)	First-line	Sorafenib (n=299)	-	10.7	-	0.69 (0.55-0.87)	-	NA	-	-	2.3	6 weeks	16	1
		Placebo (n=303)	-	7.9	-		-		-		0.7			
Faivre et al, 2009 (Phase II full text)	First-line	Sunitinib (n=37)	-	8.7	-	-	-	4	-	-	2.7	NA	NA	2
**Abou-Alfa et al, 2010 (phase II, full text)**	First-line	**Sorafenib plus Doxorubicin (n=47)**	6.5	13.7	20.1	0.49 (0.3-0.8)	2.5	6.0	9.1	0.54 (0.3-0.8	4	NA	25	3
		Doxorubicin (n=49)	-	6.5	-		-	2.7	-		2			

**Hsu et al, 2012 (phase II, full text)**	First-line	**Vandetanib 300 mg (n=19)**	3.9	5.95	9.5	0.60 (0.30-1.19)	0.98	1.05	3.6	-	0	4 weeks	12	4
		**Vandetanib 100 mg (n=25)**	4.3	5.75	-	0.44 (0.22-0.86)	0.98	1.7	3.7		0			
		Placebo (n=23)	-	4.27	-	-	-	0.95	-		0			
NCT00604721, 2012 (phase II, clinicaltrials.gov)	First-line	Selumetinib (n=19)	-	4.2	-	-	-	1.4	-	-	0	NA	NA	5
**Zhu et al, 2013 (phase II, full text)**	First-line	**Cediranib (n=17)**	6.2	11.7	13.3	-	3.2	5.3	9	-	0	8 weeks	17	6
Zhu et al, 2013 (phase II, full text)	First-line	Ramucirumab n=42	-	12	-	-	-	4	-	-	9.5	NA	NA	7
**SUN1170, 2013 (phase III full text)**	First-line	**Sorafenib (n=544)**	5	10.2	22	1.30 (1.13-1.50)	1.8	3	7	1.13 (0.99-1.30)	3.8	6 weeks	30	8
		**Sunitinib (n=530)**	4.2	7.9	16.2		1.8	3.6	5.8		4.1			
BRISK-FL*, 2013 (phase III full text)	First-line	Sorafenib (n=578)	-	9.9	-	1.07 (0.94-1.23)	-	NA	-	-	8.8	6 weeks	35	9
		Brivanib (n=577)	-	9.5	-		-		-		12			
Abou-Alfa GK et al, 2014 (phase II, full text)	First-line	Cixutumumab (n=24)	-	8	-	-	-	-	-	-	0	NA	NA	10
NCT01239355, 2015 (phase II, clinicaltrials.gov)	First-line	MK2206 (n=15)	-	6.1	-	-	-	1.7	-	-	0	NA	NA	11
Cheng et al, 2015 (phase II, full text)	First-line	Sorafenib (n=53)	-	8.2	-	-	-	NA	-	-	10.9	6 weeks	15.2	12
		Tigatuzumab (6mg/6mg) plus Sorafenib (n=54)	-	12.2	-	0.84 (0.52-1.35)	-		-		14.8			
		Tigatuzumab (6mg/2mg) plus Sorafenib (n=53)	-	8.2	-	1.24 (0.77-1.29)	-		-		5.7			
LIGHT, 2015 (phase III, full text)	First-line	Sorafenib (n=521)	-	9.8	-	1.05 (0.90-1.22)	-	2.9	-	0.81 (0.70-0.95)	6.1	6 weeks	27.7	13
		Linifanib (n=514)	-	9.1	-		-	4.2	-		10.1			
SEARCH, 2015 (phase III, full text)	First-line	Sorafenib (n=358)	-	8.5	-	0.93 (0.78-1.11)	-	NA	-	1.11 (0.94-1.31)	3.9	6 weeks	32	14
		Sorafenib plus Erlotinib (n=362)	-	9.5	-		-		-		6.6			

Ciuleanu et al, 2016 (phase II, full text)	First-line	Sorafenib plus placebo (n=51)	-	10.1	-	1.195 (0-1.651)	-	4.3	-	1.066 (0-.1430)	NA	NA	24	15
		Sorafenib plus Mapatumumab (n=50)	-	10.	-		-	3.2						
Cheng et al, 2016 (phase II, full text)	First-line	Sorafenib (n=83)	-	8.4	-	1.27 (0.90-1.79)	-	NA	-	-	10.8	6 weeks	31.9	16
		Dovitinib (n=82)	-	8.0	-		-		-		6.1			
**SARAH, 2017 (phase III, full text)**	First-line	**Sorafenib (n=222)**	5	9.9	21	1.15 (0.94-1.41)	2.3	3.7	8.3	1.03 (0.85-1.25)	10.4	4 weeks	45	17
		Y90 (n=237)	-	8	-		-	4.1	-		15.2			
**REFLECT*, 2018 (phase III, full text)**	First-line	**Sorafenib (n=476)**	5.81	12.6	25.2	0.92 (0.79-1.06)	1.83	3.7	8.7	0.66 (0.57-0.77)	24.1	8 weeks	40.5	18
		**Lenvatinib (n=478)**	6.99	13.6	27.4		3.66	7.4	16.1		9.2			
**SIRVENIB, 2018 (phase III, full text)**	First-line	**Sorafenib (n=178)**	5.2	10	21	1.10 (0.90-1.40)	2.8	5.1	9	0.89 (0.70-1.10)	1.7	12 weeks	41	19
		Y90 (n=182)	-	8.8	-		-	5.8	-		16.5			
**SILIUS*, 2018 (phase III, full text)**	First-line	**Sorafenib (n=103)**	5.9	11.5	24	1.01 (0.74-1.37)	2.4	3.5	6.2	0.5 (0.57-1.00)	17.5	NA	51.6	20
		Sorafenib + HAIC (n=(103)	-	11.8	-		-	4.8	-		36.3			
NCT02292173, 2019 (phase I, abstract)	First-line	Trametinib plus Sorafenib (n=17)	-	5.8	-	-	-	4.0	-	-	5.9	NA	NA	21
**CALGB 80802, 2019 (phase III, full text)**	First-line	**Sorafenib (n=176)**	4	9.4	15.8	1.05 (0.83-1.31)	2.31	3.7	6.7	0.93 (0.75-1.16)	NA	6 weeks	60	22
		Sorafenib plus Doxorubicin (n=180)	4	9.3	18.3		2.63	4	7.58					
Fountzilas C. et al. 2020 (phase I/II, abstract)	First-line	Tivozanib (n=27)	-	7.5	-	-	-	5.5	-	-	21	NA	NA	23
Santoro et al. 2013 (phase II, full text)	Second -line	Tivantinib (n=71)	3	6.6	17.45	0.90 (0.57-1.40)	-	1.5	-	0.67 (0.44-1.04)	1.4	6 weeks	27.8	24
		Placebo (n=36)	-	6.2	-		-	1.4	-		0			
**BRISK-PS*, 2013 (phase III, full text)**	Second -line	Brivanib (n=263)	-	9.4	-	0.89 (0.69-1.15)	-	NA	-	-	9.9	6 weeks	30	25
		Placebo (n=132)	-	8.2	-		-		-		1.5			
**EVOLVE-1, 2014 (phase III, full text)**	Second -line	Everolimus (n=362)	-	7.6	-	1.05 (0.86-1.27)	-	NA	-	-	3	6 weeks	22	26

**Kang et al, 2015 (phase II, full text)**	Second -line	**Axitinib (n=134)**	5.9	12.7	22.1	0.91 (0.65-1.27)	1.9	3.6	7.5	0.62 (0.44-0.87)	9.7	8 weeks	34	27
		Placebo (n=68)	-	9.7	-		-	1.9	-		2.9			
**REACH, 2015 (phase III, full text)**	Second -line	**Ramucirumab (n=283)**	4.85	9.2	21.75	0.87 (0.72-1.05)	1.59	2.8	7.6	0.63 (0.52-0.75)	7.1	6 weeks	37	28
		Placebo (n=282)	-	7.6	-		-	2.1	-		0.7			
NCT01488487, 2016 (phase II, clinicaltrials.gov)	Second -line	Everolimus plus Pasireotide (n= 24)	-	6.7	-	-	-	NA	-	-	0	NA	NA	29
**Abou-Alfa et al, 2016 (phase II, full text)**	Second -line	**Codrituzumab (n=125)**	3.75	8.7	14.8	0.96 (0.65-1.41)	1.3	2.6	5.6	0.97	NA	6 weeks	19.5	30
		Placebo (n=60)	-	10	-		-	1.5	-					
**Escudier et al, 2017 (phase II, full text)**	Second -line	**Tasquinimod (n= 53)**	5.8	7.3	14.8	-	2.1	3.9	6.5	-	1.9	NA	13.8	31
**RESORCE*, 2017 (phase III, full text)**	Second -line	**Regorafenib (n=379)**	4.88	10.6	20.13	0.63 (0.50-0.79)	1.88	3.1	7.65	0.46 (0.37-0.56)	3.2	6 weeks	33	32
		Placebo (n=194)	-	7.8	-		-	1.5	-		1.5			
**METIV-HCC, 2018 (phase III, full text)**	Second -line	**Tivantinib (n=226)**	4.1	8.4	17.2	0.97 (0.75-1.25)	1.8	2.1	4.75	0.96 (0.75-1.22)	0.0	8 weeks	36	33
		Placebo (n=114)		9.1			-	2	-		0.0			
**Abou-Alfa et al, 2018 (phase III, full text)**	Second -line	**ADI-PEG20 (n=424)**	3.8	7.8	14.9	1.02 (0.85-1.23)	2.45	2.6	5.1	1.18 (0.96-1.43)	NA	12 weeks	48	34
		Placebo (n=211)	-	7.4	-		2.45	2.6	5.35					
**CELESTIAL, 2018 (phase III, full text)**	Second -line	**Cabozantinib (n=470)**	6.02	10.2	19.9	0.76 (0.63-0.92)	1.97	5.2	8.99	0.44 (0.36-0.52)	5.4	8 weeks	41	35
		Placebo (n=237)	-	8	-		-	1.9	-		1.9			
**REACH-2, 2019 (phase III, full text)**	Second -line	**Ramucirumab (n=197)**	4.5	8.5	16.8	0.71 (0.53-0.95)	1.5	2.8	5.8	0.45 (0.34-0.60)	4.6	6 weeks	27	36
		Placebo (n=95)	-	7.3	-		-	1.6	-		1.61			
Philip et al, 2012 (phase II, full text)	Both first- and second-line	Bevacizumab plus Erlotinib n= 27	5.9	9.5	21.5	-	-	NA	-	-	4.3	8 weeks	36	37

The treatment arms from which the individual data for overall survival and progression-free survival were extracted are shown in bold.

All the included trials employed RECIST 1.1 criteria, except those marked with *, that employed mRECIST criteria.

HR, Hazard Ratio. 95% CI, 95% Confidence intervals. NA, not available.

Table S3. Test of proportionality of hazards between PFS and OS in reconstructed survival curves for immune checkpoint inhibitors (ICIs) and multikinase inhibitors (MKIs).

Class of drug	Trial arm	Line of treatment	Chi-square	P-value
Immune checkpoint inhibitors	IMBrave-150 (Atezolizumab plus Bevacizumab arm)	First-Line	6.69	0.0097
	CheckMate 459 (Nivolumab arm)	First-Line	21.9	$2.83*10^{-6}$
	Keynote-224 (Pembrolizumab arm)	Second-line	3.17	0.075
	Keynote-240 (Pembrolizumab arm)	Second-line	26.8	$2.26*10^{-7}$
	Qin et al., 2020 (Camrelizumab arm)	Second-line	12.8	0.00034
	CheckMate 040 (Nivolumab plus Cabozantinib arm)	Both first- and second-line	1.7	0.193
	CheckMate 040 (Nivolumab plus Cabozantinib plus Ipilimumab arm)	Both first- and second-line	2.11	0.146
**Pooled immune checkpoint inhibitors**			85.8	$2.04 * 10^{-20}$
Multikinase inhibitors	SUN1170 (Sorafenib arm)	First-line	13.8	0.0002
	SUN1170 (Sunitinib arm)	First-line	5.19	0.023
	SARAH (Sorafenib arm)	First-line	5.81	0.016
	SIRVENIB (Sorafenib arm)	First-line	0.075	0.785
	CALGB80802 (Sorafenib arm)	First-line	7.47	0.0063
	SILIUS (Sorafenib arm)	First-line	9.81	0.002
	IMBrave-150 (Sorafenib arm)	First-line		
